# Rare Co-occurrence of Beta-Thalassemia and *Pseudoxanthoma elasticum*: Novel Biomolecular Findings

**DOI:** 10.3389/fmed.2019.00322

**Published:** 2020-01-23

**Authors:** Federica Boraldi, Francesco Demetrio Lofaro, Sonia Costa, Pasquale Moscarelli, Daniela Quaglino

**Affiliations:** Department of Life Sciences, University of Modena and Reggio Emilia, Modena, Italy

**Keywords:** genetic testing, beta-thalassemia, PXE, *ABCC6*, *ENPP1*

## Abstract

A number of beta-thalassemia patients, independently from the type of beta-thalassemia (β^0^ or β^+^) and blood transfusion requirements, may develop, after puberty, dermal, cardiovascular, and ocular complications associated with an ectopic mineralization phenotype similar to that observed in another rare genetic disorder, namely, *Pseudoxanthoma elasticum* (PXE). To date, the causes of these alterations in beta-thalassemia patients are not known, but it has been suggested that they could be the consequence of oxidative stress-driven epigenetic regulatory mechanisms producing an *ABCC6* down-regulation. Since, in the last years, several genes have been associated to the ectopic mineralization phenotype, this study, for the first time, applied, on beta-thalassemia patients with ectopic mineralization phenotype, a multigene testing strategy. Selection of genes to be analyzed was done on the basis of (i) their genetic involvement in calcification diseases or (ii) their role in calcium-phosphate equilibrium. Although, due to the rarity of these conditions, a limited number of patients was analyzed, the detection of pathogenic variants represents the proof of concept that PXE and beta-thalassemia traits co-occur on a genetic basis and that, in addition to causative mutations, functional polymorphisms may further influence connective tissue manifestations. The use of a multigene-based next-generation sequencing represents a useful time- and cost-effective approach, allowing to identify sequence variants that might improve prognostic assessment and better management of these patients, especially in the current era of precision medicine aiming to identify individual optimal care based on a unique personal profile.

## Introduction

Beta-thalassemia (β-thal) (OMIM 613985) is a genetic disorder caused by mutations in the β-hemoglobin (*HBB*) gene that determines either a reduced (β^+^) or absent (β^0^) synthesis of the β-globin chains. In addition to hematological findings, according to the literature, up to 10% of β-thal patients (1) exhibit clinical manifestation similar to those observed in *Pseudoxanthoma elasticum* (PXE) (OMIM 264800), a rare genetic disease in which *ABCC6* gene mutations cause the mineralization of elastic fibers responsible for skin, ocular, and cardiovascular complications that generally start after puberty and continuously progress with age (2).

A first study failed to detect *ABCC6* gene mutations in 10 β-thal patients with soft connective tissue complications (3) and in the light of these data, the involvement, in these patients, of *ABCC6* gene mutations in the pathogenesis of PXE-like alterations was ruled out. Therefore, PXE-like manifestations in β-thal patients have been hypothesized to be the consequence of oxidative stress-driven epigenetic regulatory mechanisms causing *ABCC6* down-regulation (4). However, in the recent years, the expanding knowledge on the mechanisms controlling the mineralization process has revealed that, in addition to *ABCC6*, several other genes can be responsible for ectopic mineralization phenotypes (2), but, to our knowledge, a multigene approach has not been applied to patients in which beta-thalassemia is associated with PXE-like connective tissue manifestation.

The aim of this study was to define a multigene next-generation sequencing to identify pathogenic variants in three unrelated β-thal patients exhibiting soft connective tissue manifestations.

## Materials and Methods

### Patients

**Figure 1:**
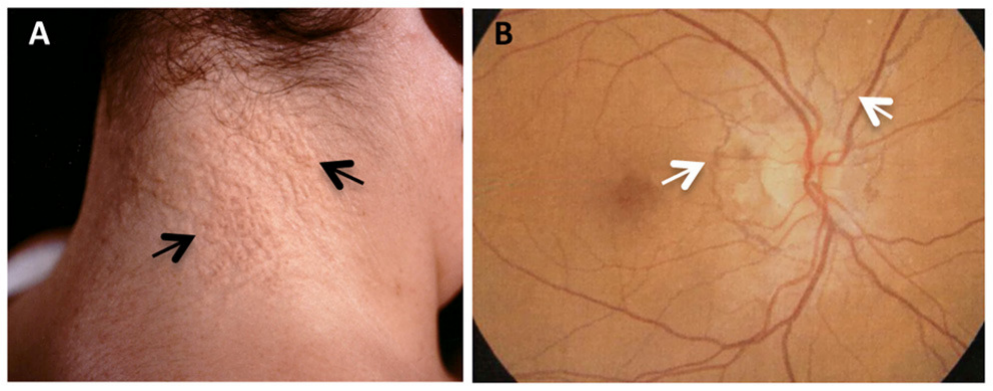
PXE clinical features in beta-thalassemia patients. **(A)** Skin papules (arrows) typically located on the neck; **(B)** fluoroangiography showing angioid streaks (arrows) as irregular lines spreading from the optic nerve to the retinal periphery.

In the present study, three unrelated beta-thalassemia female patients with skin papules and/or skin laxity typical of PXE (Figure 1A) and by ocular angioid streaks (Figure 1B) have been investigated.

The diagnosis of beta-thalassemia was based on the usual hematological criteria (peripheral blood evaluation and hemoglobin electrophoresis) and on the presence of mutations in *HBB* gene. Two patients (#1 and #3) were diagnosed with β-thalassemia Major and were carrier of a pathogenic variant in intron 1 (IVS1-110G>A in the homozygous state) and a stop codon mutation in exon 2 (c.C118T, p.Gln40^*^ in the homozygous state), respectively. Both patients required blood transfusion and chelation therapy. The third patient (#2) was diagnosed with β-thalassemia and was carried of a pathogenic variant in intron 1 (IVS1-110G>A in the heterozygous condition). This patient never required blood transfusion.

Clinical laboratory tests (i.e., Ca, P, intact PTH, and ALPL) were within normal ranges.

The study was done, with informed consent, in accordance with the basic principles of the Declaration of Helsinki and approval by the local Ethical Committee (Comitato Etico Provinciale di Modena) (n. 358/17).

### Targeted Exome Sequencing

DNA was extracted from whole blood using standard methods and quantified and quality tested using the Qubit 2.0 Fluorometer (Invitrogen, Carlsbad, CA) and Agilent 2100 Bioanalyzer (Agilent Technologies, Santa Clara, CA). Samples were subjected to target exome sequencing. A DNA custom panel was designed with AmpliSeq Custom DNA Panel for Illumina and sequenced on NextSeq500 (Illumina).

Raw reads were quality trimmed at both ends with ERNE-filter v1.4.3 (5) using default parameters and minimum read length of 40 bp.

Reads were aligned to the human reference genome (GRCh37/hg19 assembly) and only reads, mapping to unique positions, were retained. Variant discovery and functional annotation were performed by the software tool ANNOVAR (6).

**Figure 2:**
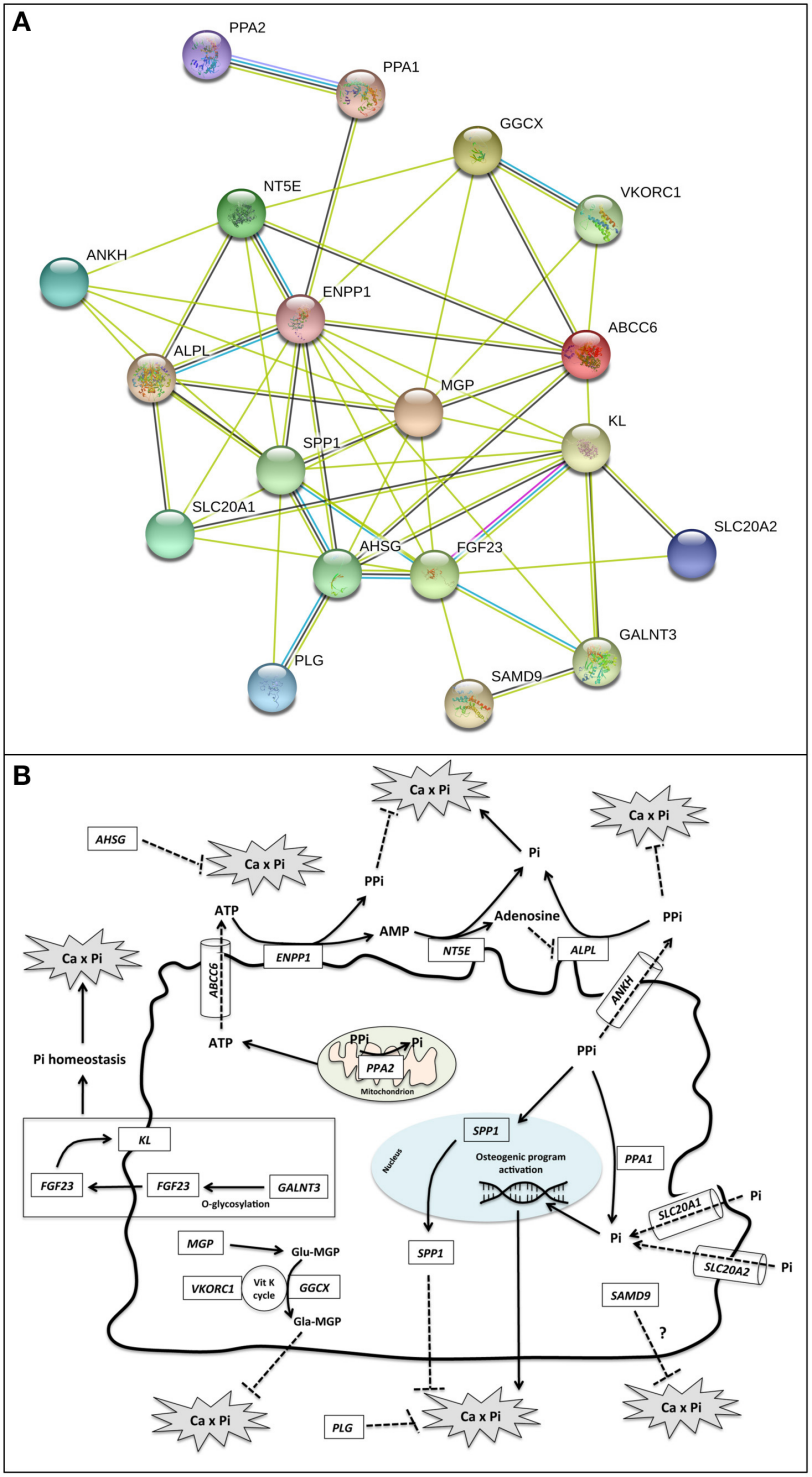
**(A)** Gene–protein interaction network. Gene relationships were obtained by STRING 11 (Search Tool for the Retrieval of Interacting Genes/Proteins) software. STRING parameters included: Active Prediction Methods: Textmining (yellow), Experiments (violet), Databases (light blue), and Co-expression (black); max number of interactors: none. *ABCC6* = Multidrug resistance-associated protein 6; *ALPL* = Alkaline phosphatase, tissue-non-specific isozyme; *AHSG*, Alpha-2-HS-glycoprotein; *ANKH*, Progressive ankylosis protein homolog; *ENPP1*, Ectonucleotide pyrophosphatase/phosphodiesterase family member 1; *FGF23*, Fibroblast growth factor 23; GALNT3, Polypeptide N-acetylgalactosaminyltransferase 3; *GGCX*, Vitamin K-dependent gamma-carboxylase; *KL*, Klotho; MGP = Matrix Gla protein; *NT5E*, 5'-nucleotidase; *PLG*, Plasminogen; *PPA1*, Inorganic pyrophosphatase; *PPA2*, Inorganic pyrophosphatase 2; *SAMD9*, Sterile alpha motif domain-containing protein 9; *SLC20A1*, Sodium-dependent phosphate transporter 1; *SLC20A2*, Sodium-dependent phosphate transporter 2; *SPP1*, Osteopontin; *VKORC1*, Vitamin K epoxide reductase complex subunit 1. **(B)** Schematic representation of functional roles of calcification-related genes. Localization, substrate specificity, and/or transported molecules are reported for the investigated genes.

Analyses were performed focusing on a panel of 19 genes, 12 of them are responsible for calcification-related diseases and 7 of them play a role in calcium-phosphate equilibrium (7–13) (Figures 2A,B and Table S1).

#### Filtering of Genetic Variants

Analyses were carried out primarily looking at coding and splice site regions excluding reference sequences and variants that do not change the translated amino acid sequence (i.e., synonymous). Thereafter, the frequency of each sequence variant was assessed querying databases as dbSNP138 build, 1,000 Genomes Project (1000g2012apr_all), and ESP6500si_all in order to discriminate common variants, with a minor allele frequency ≥1%, and rare sequence variants.

#### In Silico Analysis for Functional Prediction of Variants

For rare sequence variants not yet reported in the literature as pathogenic, the pathogenicity score was predicted by Sorting Intolerant From Tolerant (SIFT) (14), Polymorphism Phenotyping (Polyphen2) (15), and Panther (16), whereas the Genomic Evolutionary Rate Profiling (GERP++) (17) method and the Phylogenetic P-values (PhyloP) (18) were used to estimate the level of single-nucleotide variation conservation. To predict protein stability I-Mutant and MUpro were used (19).

### Sanger Sequencing

All pathogenic variants were confirmed by conventional Sanger sequencing. Primer sequences were designed by NCBI Primer-BLAST software, and purified PCR products were directly sequenced using an Applied Biosystems 3100 DNA sequencer (20).

## Results and Discussion

Unexpectedly, rare sequence variants were detected in *ABCC6* and *ENPP1* genes (Table 1 and Figure S1).

Table 1Clinical/demographic data of patients (#1, #2, and #3) and rare sequence variants found in *HBB, ABCC6*, and *ENPP1* genes.

**Table:** 

**ID (Age/Gender)**	**Rare sequence variants**					
	***HBB***		***ABCC6***		***ENPP1***	
	**Allele 1**	**Allele 2**	**Allele 1**	**Allele 2**	**Allele 1**	**Allele 2**
**#1** (28/F)	IVS1-110G>A	IVS1-110G>A	c.1171A>G p.Arg391Gly	c.1171A>G p.Arg391Gly	–	–
**#2** (31/F)	IVS 1-110G>A	–	**c.4055T>C** **p.Phe1352Ser**	c.3340C>T p.Arg1114Cys	–	–
**#3** (27/F)	c.118C>T, p.Gln40^*^	c.118C>T, p.Gln40^*^	c.1171A>G p.Arg391Gly	–	**c.2657G>C** **p.Arg886Thr**	–

**Table:** 

**Characteristic of rare sequence variants reported in this study**						
**Gene symbol**	**Variant**	**Genomic coordinates** **(GRCh37)**	**Exon/Intron**	**dbSNP ID**	**Frequency** **1,000G**
*HBB*	IVS1-110G>A	chr11: 5248050	Intron1	rs35004220	NA
	c.118C>T	chr11: 5248004	Exon2	rs11549407	0.000
*ABCC6*	c.1171A>G	chr16: 16295863	Exon9	rs72653762	0.003
	c.3340C>T	chr16: 16257016	Exon24	rs63749794	0.000
	c.4055T>C	chr16: 16248638	Exon29	NA	NA
*ENPP1*	c.2657G>C	chr6: 132211530	Exon25	rs8192683	0.003

*New rare sequence variants are highlighted in bold*.

*NA, not available*.

*ABCC6* is well-known as a PXE causing gene, encoding for a transmembrane protein (MRP6) probably involved in the extrusion of ATP (Figure 2B) (21).

*ENPP1* encodes for an ectonucleotide-pyrophosphatase/phosphodiesterase-1 that utilizes adenosine triphosphate to release pyrophosphate (PPi) (2, 22), a potent inhibitor of ectopic calcification (Figure 2B).

Note that *ABCC6* and *ENPP1* have been both recently recognized as PXE causing genes (22).

Patient #1 was homozygous for *ABCC6*_*Arg391Gly*_ (22), patient #2 was compound heterozygous for *ABCC6*_*Phe1352Ser*_ and *ABCC6*_*Arg1114Cys*_ (22), and patient #3 was heterozygous for *ABCC6*_*Arg391Gly*_ and for ENPP1_*Arg886Thr*_ (Table 1).

Of these variants, *ABCC6*_*Phe1352Ser*_ and ENPP1_*Arg886Thr*_ were never found in the literature, but, for both missense variants, the prediction scores implied a probably damaging effect (Table S2).

Present data underline the importance of *ABCC6* and also provide evidence that, in one patient, an *ABCC6* pathogenic allele is associated with a new rare sequence variant in the *ENPP1* gene (c.2657G > C, p.Arg886Thr), where a substitution of the highly conserved arginine in position 886 with a threonine can affect the structural stability and the functional properties of the protein due to different polarity, charges, and size of these two amino acids. In the light of these data, a digenic inheritance of PXE manifestation could be suggested, as already shown for *ABCC6* and *GGCX* (23).

Sequence variants were never detected in the following genes: *AHSG, ANKH, GALNT3, KL, PLG, PPA1, SLC20A1, SLC20A2*, and *VKORC1*. However, the absence of rare sequence variants and/or single-nucleotide polymorphisms (SNPs) in these genes does not exclude *a priori* their possible role in the development of ectopic mineralization phenotypes, and therefore, in perspective, studies on a larger cohort of patients would better elucidate this aspect.

SNPs were detected in other genes (Table 2 and Table S3).

**Table 2:** Single-nucleotide polymorphisms (SNPs) found in at least one patient.

**Gene**	**Common sequence variants**						
	**SNP ID**	**Nucleotide change**	**Amino acid change**	**Genotypes**		
				**#1**	**#2**	**#3**
*ABCC6*	rs2238472	c.3803G>A	Arg1268Gln	AA	GG	GA
*ALPL*	rs3200254	c.787T>C	Tyr263His	TT	TT	TC
*ENPP1*	rs1044498	c.517A>C	Lys173Gln	CC	AC	AC
*FGF23*	rs7955866	c.716C>T	Thr239Met	CC	CC	CT
*GGCX*	rs699664	c.974G>A	Arg325Gln	GG	GA	GA
*MGP*	rs4236	c.304A>G	Thr102Ala	AG	AA	AG
*NT5E*	rs2229523	c.1126A>G	Thr376Ala	GG	GG	GG
*PPA2*	rs13787	c.846G>C	Lys282Asn	CC	GG	GG
*SAMD9*	rs10239435 rs6969691	c.1346A>G c.428T>C	Asn449Ser Ile143Thr	AA TT	AA TT	AG TC
*SPP1*	rs11728697	c.71C>T	Ala24Val	CC	TT	TT

Interestingly, two of these polymorphisms have been reported in the literature to modulate the ectopic mineralization phenotype: rs1044498 in *ENPP1* (24) and rs7955866 in *FGF23* (25).

*ENPP1*_Lys173Gln_ is a functional polymorphism found in all three patients in the homozygous (#1) or heterozygous state (#2 and #3). This polymorphism (c.517A>C; p.Lys173Gln) has been demonstrated to reduce enzyme activity, thus causing diminished calcification inhibitory properties of inorganic pyrophosphate (PPi) (24) and consequently predisposing to cardiovascular complications as coronary calcification and earlier onset of peripheral arterial disease.

Moreover, it could be suggested that rare sequence variants and/or functional polymorphisms in *ENPP1* can act in synergy with epigenetic regulatory mechanisms. The oxidative stress condition, in fact, can down-regulate, by epigenetic mechanisms, the activity of *ENPP1* (26) and this finding is of special interest in β-thal patients who are known to be characterized by an altered redox balance (27).

As far as *FGF23*_*Thr239Met*_, this SNP, found in patient #3 in the heterozygous condition, has been reported to influence *FGF23* biological function, providing an increased stability of the protein, thus influencing phosphate homeostasis and favoring kidney stone formation (25).

Interestingly, the *ABCC6*_*Arg1268Gln*_ sequence variant was detected in two patients, in the homozygous (#1) and in the heterozygous state (#3). Although considered in early studies to be pathogenic, given its frequency (19%), it has been later considered as a functional polymorphism contributing to the development and/or severity of angioid streaks (28, 29) and therefore cannot be disregarded in a prognostic assessment.

As far as rs699664 in *GGCX*, rs4236 in *MGP*, and rs2229523 in *NT5E* genes, data in the literature (30–32) indicate that SNPs observed in our patients determine either an enzyme activity similar to wildtype *(GGCX*_*Arg325Gln*_ and *NT5E*_*Thr376Ala*_*)* or absent association with increased risk of calcification *(MGP*_*Thr102Ala*_*)* and therefore their functional contribution to the ectopic mineralization phenotype can be likely irrelevant.

To our knowledge, other SNPs found in *ALPL* (rs3200254), *PPA2* (rs13787), *SAMD9* (rs10239435 and rs6969691), and *SSP1* (rs11728697) genes have not been investigated for their possible functional role, and therefore, at present, pathogenic hypotheses cannot be put forward.

Evidence is provided that there are patients in which, independently from the type of beta-thalassemia (β^0^ or β^+^) and blood transfusion requirements, PXE clinical manifestations can occur on a genetic basis.

These data are partially in contrast to previous observations suggesting the absence of *ABCC6* pathogenic variants in β-thal patients (3). This discrepancy could be due to (i) the continuously increasing number of recognized pathogenic variants (>350) since the discovery of *ABCC6* as the causative PXE gene and (ii) the demonstrated pathogenic role of other genes in addition to *ABCC6* (2, 22).

## Conclusion

Considering the current longer life expectancy of β-thal patients, the development of complications that appear and progress with age should deserve higher consideration in both clinical and genetic evaluations. In the light of present data, searching for sequence variants in calcification-related genes also at the pre-symptomatic stage is recommended for a better management of β-thal patients and of their possible complications. Moreover, although this condition may affect a limited number of patients, genetic tests, given their increasing widespread distribution, can easily provide high-throughput and informative data to make a more accurate diagnostic and prognostic assessment, especially in the current era of precision medicine, distinguishing a given patient from other patients with similar clinical phenotype.

## Data Availability Statement

All datasets for this study are included in the article/Supplementary Material.

## Ethics Statement

The studies involving human participants were reviewed and approved by Comitato Etico Provinciale di Modena. The patients/participants provided their written informed consent to participate in this study.

## Author Contributions

FB designed the study and wrote the manuscript. FL, SC, and PM collected samples and performed the research. FB and FL analyzed the results. DQ supervised the research and critically revised the manuscript. All authors approved the final submitted version of the manuscript.

### Conflict of Interest

The authors declare that the research was conducted in the absence of any commercial or financial relationships that could be construed as a potential conflict of interest.
