# Supplementary material for: Rare Co-occurrence of Beta-Thalassemia and Pseudoxanthoma elasticum: Novel Biomolecular Findings
Source: Front Med (Lausanne). 2020 Jan 23;6:322. doi: 10.3389/fmed.2019.00322 (PMC6989569; doi:10.3389/fmed.2019.00322)
Supplement: Supplementary file 1 [file Data_Sheet_1.PDF]

Supplementary Appendix

Rare co-occurrence of beta-thalassemia and Pseudoxanthoma elasticum: novel biomolecular findings.

Federica Boraldi, Francesco Demetrio Lofaro, Sonia Costa, Pasquale Moscarelli, Daniela Quaglino.

Department of Life Sciences, University of Modena and Reggio Emilia, Via Campi 287, 41125 Modena (Italy)

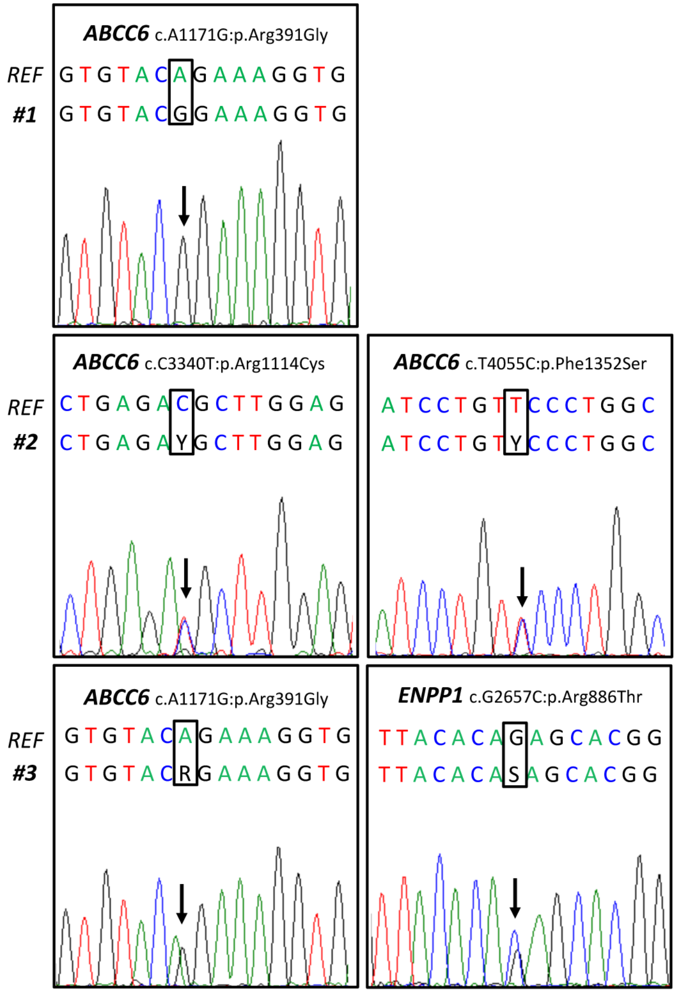

Supplemental Figure 1 Sanger sequencing electropherograms. Homozygous (#1) and heterozygous (#2 and #3) rare *ABCC6* and *ENPP1* sequence variants were detected in three beta-thal patients. REF: DNA reference sequence; #1, #2, #3: DNA sequence in each patient.

Supplemental Table 1. Calcification-related multi-gene panel. The selection of the genes was done on the basis of their involvement in calcification-related genetic diseases or in calcium-phosphate equilibrium. In bold are rare sequence variants found in at least one patient. Genes highlighted in grey do not show sequence variants.

	Gene/ Protein	Protein name	RefSeq Summary
1	**ABCC6/ MRP6**	**Multidrug resistance-associated protein 6**	The protein encoded by this gene is a member of the superfamily of ATP-binding cassette (ABC) transporters. ABC proteins transport various molecules across extra- and intra-cellular membranes. ABC genes are divided into seven distinct subfamilies (ABC1, MDR/TAP, MRP, ALD, OABP, GCN20, White). The encoded protein, a member of the MRP subfamily, is involved in multi-drug resistance. Mutations in this gene cause pseudoxanthoma elasticum. Alternatively, spliced transcript variants that encode different proteins have been described for this gene. [provided by RefSeq, Jul 2008].
2	ALPL/ PPBT	Alkaline phosphatase, tissue-nonspecific isozyme	This gene encodes a member of the alkaline phosphatase family of proteins. There are at least four distinct but related alkaline phosphatases: intestinal, placental, placental-like, and liver/bone/kidney (tissue non-specific). The first three are located together on chromosome 2, while the tissue non-specific form is located on chromosome 1. The product of this gene is a membrane bound glycosylated enzyme that is not expressed in any particular tissue and is, therefore, referred to as the tissue-nonspecific form of the enzyme. Alternative splicing results in multiple transcript variants, at least one of which encodes a preproprotein that is proteolytically processed to generate the mature enzyme. This enzyme may play a role in bone mineralization. Mutations in this gene have been linked to hypophosphatasia, a disorder that is characterized by hypercalcemia and skeletal defects. [provided by RefSeq, Oct 2015].
3	AHSG/ FETUA	Alpha-2-HS-glycoprotein	The protein encoded by this gene is a negatively-charged serum glycoprotein that is synthesized by hepatocytes. The encoded protein consists of two polypeptide chains, which are both cleaved from a proprotein encoded from a single mRNA. It is involved in several processes, including endocytosis, brain development, and the formation of bone tissue. Defects in this gene are a cause of susceptibility to leanness. [provided by RefSeq, Aug 2017]
4	ANKH/ ANKH	Progressive ankylosis protein homolog	This gene encodes a multipass transmembrane protein that is expressed in joints and other tissues and controls pyrophosphate levels in cultured cells. Progressive ankylosis-mediated control of pyrophosphate levels has been suggested as a possible mechanism regulating tissue calcification and susceptibility to arthritis in higher animals. Mutations in this gene have been associated with autosomal dominant craniometaphyseal dysplasia. [provided by RefSeq, Jul 2008]
5	**ENPP1/ ENPP1**	**Ectonucleotide pyrophosphatase/phosphodiesterase family**	This gene is a member of the ecto-nucleotide pyrophosphatase/phosphodiesterase (ENPP) family. The encoded protein is a type II transmembrane glycoprotein comprising two identical disulfide-

		**member 1**	bonded subunits. This protein has broad specificity and cleaves a variety of substrates, including phosphodiester bonds of nucleotides and nucleotide sugars and pyrophosphate bonds of nucleotides and nucleotide sugars. This protein may function to hydrolyze nucleoside 5' triphosphates to their corresponding monophosphates and may also hydrolyze diadenosine polyphosphates. Mutations in this gene have been associated with 'idiopathic' infantile arterial calcification, ossification of the posterior longitudinal ligament of the spine (OPLL), and insulin resistance. [provided by RefSeq, Jul 2008]
6	FGF23/ FGF23	Fibroblast growth factor 23	This gene encodes a member of the fibroblast growth factor family of proteins, which possess broad mitogenic and cell survival activities and are involved in a variety of biological processes. The product of this gene regulates phosphate homeostasis and transport in the kidney. The full-length, functional protein may be deactivated via cleavage into N-terminal and C-terminal chains. Mutation of this cleavage site causes autosomal dominant hypophosphatemic rickets (ADHR). Mutations in this gene are also associated with hyperphosphatemic familial tumoral calcinosis (HFTC). [provided by RefSeq, Feb 2013]
7	GALNT3/ GALT3	Polypeptide N-acetylgalactosaminyltransferase 3	This gene encodes UDP-GalNAc transferase 3, a member of the GalNAc-transferases family. This family transfers an N-acetyl galactosamine to the hydroxyl group of a serine or threonine residue in the first step of O-linked oligosaccharide biosynthesis. Individual GalNAc-transferases have distinct activities and initiation of O-glycosylation is regulated by a repertoire of GalNAc-transferases. The protein encoded by this gene is highly homologous to other family members, however the enzymes have different substrate specificities. [provided by RefSeq, Jul 2008]
8	GGCX/ VKGC	Vitamin K-dependent gamma-carboxylase	This gene encodes an integral membrane protein of the rough endoplasmic reticulum that carboxylates glutamate residues of vitamin K-dependent proteins to gamma carboxyl glutamate, a modification that is required for their activity. The vitamin K-dependent protein substrates have a propeptide that binds the enzyme, with carbon dioxide, dioxide, and reduced vitamin K acting as co-substrates. Vitamin K-dependent proteins affect a number of physiologic processes including blood coagulation, prevention of vascular calcification, and inflammation. Allelic variants of this gene have been associated with pseudoxanthoma elasticum-like disorder with associated multiple coagulation factor deficiency. Alternative splicing results in multiple transcript variants. [provided by RefSeq, Aug 2015]
9	KL/ KLOT	Klotho	This gene encodes a type-I membrane protein that is related to beta-glucosidases. Reduced production of this protein has been observed in patients with chronic renal failure (CRF), and this may be one of the factors underlying the degenerative processes (e.g., arteriosclerosis, osteoporosis, and skin atrophy) seen in CRF. Also, mutations within this protein have been associated with ageing and bone loss. [provided by RefSeq, Jul 2008]
10	MGP/ MGP	Matrix Gla protein	This gene encodes a member of the osteocalcin/matrix Gla family of proteins. The encoded vitamin K-dependent protein is secreted by chondrocytes and vascular smooth muscle cells, and functions as a physiological inhibitor of ectopic tissue calcification. Carboxylation status of the encoded protein is associated with calcification of the vasculature in human patients with cardiovascular disease and calcification of the synovial membranes in osteoarthritis patients. Mutations in this gene cause

			Keutel syndrome in human patients, which is characterized by abnormal cartilage calcification, peripheral pulmonary stenosis and facial hypoplasia. [provided by RefSeq, Sep 2016]
11	NT5E/ 5NTD	5'-nucleotidase	The protein encoded by this gene is a plasma membrane protein that catalyzes the conversion of extracellular nucleotides to membrane-permeable nucleosides. The encoded protein is used as a determinant of lymphocyte differentiation. Defects in this gene can lead to the calcification of joints and arteries. Two transcript variants encoding different isoforms have been found for this gene.[provided by RefSeq, Mar 2011]
12	PLG/ PLMN	Plasminogen	The protein encoded by this gene is a secreted blood zymogen that is activated by proteolysis and converted to plasmin and angiostatin. Plasmin dissolves fibrin in blood clots and is an important protease in many other cellular processes while angiostatin inhibits angiogenesis. Defects in this gene are likely a cause of thrombophilia and ligneous conjunctivitis. Two transcript variants encoding different isoforms have been found for this gene.[provided by RefSeq, Dec 2009]
13	PPA1/ IPYR	Inorganic pyrophosphatase	The protein encoded by this gene is a member of the inorganic pyrophosphatase (PPase) family. PPases catalyze the hydrolysis of pyrophosphate to inorganic phosphate, which is important for the phosphate metabolism of cells. Studies of a similar protein in bovine suggested a cytoplasmic localization of this enzyme. [provided by RefSeq, Jul 2008]
14	PPA2/ IPRK2	Inorganic pyrophosphatase 2	The protein encoded by this gene is localized to the mitochondrion, is highly similar to members of the inorganic pyrophosphatase (PPase) family, and contains the signature sequence essential for the catalytic activity of PPase. PPases catalyze the hydrolysis of pyrophosphate to inorganic phosphate, which is important for the phosphate metabolism of cells. Alternate transcriptional splice variants, encoding different isoforms, have been characterized. [provided by RefSeq, Jul 2008]
15	SAMD9/ SAMD9	Sterile alpha motif domain-containing protein 9	This gene encodes a sterile alpha motif domain-containing protein. The encoded protein localizes to the cytoplasm and may play a role in regulating cell proliferation and apoptosis. Mutations in this gene are the cause of normophosphatemic familial tumoral calcinosis. Alternate splicing results in multiple transcript variants that encode the same protein.[provided by RefSeq, Jul 2010]
16	SLC20A1/ S20A1	Sodium-dependent phosphate transporter 1	The protein encoded by this gene is a sodium-phosphate symporter that absorbs phosphate from interstitial fluid for use in cellular functions such as metabolism, signal transduction, and nucleic acid and lipid synthesis. The encoded protein is also a retroviral receptor, causing human cells to be susceptible to infection by gibbon ape leukemia virus, simian sarcoma-associated virus, feline leukemia virus subgroup B, and 10A1 murine leukemia virus.[provided by RefSeq, Mar 2011]
17	SLC20A2/ S20A2	Sodium-dependent phosphate transporter 2	This gene encodes a member of the inorganic phosphate transporter family. The encoded protein is a type 3 sodium-dependent phosphate symporter that plays an important role in phosphate homeostasis by mediating cellular phosphate uptake. The encoded protein also confers susceptibility to viral infection as a gamma-retroviral receptor. Mutations in this gene may play a role in familial idiopathic basal ganglia calcification. Alternatively, spliced transcript variants encoding multiple isoforms have been observed for this gene. [provided by RefSeq, Mar 2012]
18	SPP1/ OSTP	Osteopontin	The protein encoded by this gene is involved in the attachment of osteoclasts to the mineralized bone matrix. The encoded protein is secreted and binds hydroxyapatite with high affinity. The osteoclast vitronectin receptor is found in the cell membrane and may be involved in the binding to

			this protein. This protein is also a cytokine that upregulates expression of interferon-gamma and interleukin-12. Several transcript variants encoding different isoforms have been found for this gene. [provided by RefSeq, Oct 2011]
19	VKORC1/ VKOR1	Vitamin K epoxide reductase complex subunit 1	This gene encodes the catalytic subunit of the vitamin K epoxide reductase complex, which is responsible for the reduction of inactive vitamin K 2,3-epoxide to active vitamin K in the endoplasmic reticulum membrane. Vitamin K is a required co-factor for carboxylation of glutamic acid residues by vitamin K-dependent gamma-carboxylase in blood-clotting enzymes. Allelic variation in this gene is associated with vitamin k-dependent clotting factors combined deficiency of 2, and increased resistance or sensitivity to warfarin, an inhibitor of vitamin K epoxide reductase. Pseudogenes of this gene are located on chromosomes 1 and X. Alternative splicing results in multiple transcript variants. [provided by RefSeq, Aug 2015]

Supplemental Table 2. *In silico* analysis for the pathogenicity of new missense rare sequence variants found in *ABCC6* and *ENPP1* genes.

Gene/Protein sequence variants	*Severity of aminoacid substitution*			*Evolutionary conservation*		*Protein stability*	
	**Polyphen2**[a] (score)	**SIFT**[b] (score)	**Panther**[a] (preservation time)	**PhyloP** (score)	**GERP++** (score)	**MUpro** (DDG)	**I-Mutant** (Reliability Index)
ABCC6 c.4055T>C p.Phe1352Ser	D 1	D 1	D 910	C 0.97	3,55	Decrease stability (-1.55)	Decrease stability (7)
ENPP1 c.2657G>C p.Arg886Thr	B 0	D 1	D 455	C 0.99	3.55	Decrease stability (-0.83)	Decrease stability (8)

a) D = probably damaging; B = probably benign
b) D = deleterious

Supplemental Table 3. Genetic variants in gene coding regions found in the present study

#CHROM	POS	ID	REF	ALT	FILTER	dbSNP138	1000g
chr1	21894735	ALPL	T	C	.	rs3200254	0,28
chr2	85780536	GGCX	C	T	.	rs699664	0,41
chr4	106317429	PPA2	C	G	.	rs13787	0,45
chr4	88898941	SPP1	C	T	.	rs11728697	0,42
chr6	132172368	ENPP1	A	C	.	rs1044498	0,29
chr6	132211530	ENPP1	G	C	.	rs8192683	0,0009
chr6	86199233	NT5E	A	G	.	rs2229523	0,76
chr7	92734065	SAMD9	T	C	.	rs10239435	0,05
chr7	92734983	SAMD9	A	G	.	rs6969691	0,17
chr12	4479549	FGF23	G	A	.	rs7955866	0,14
chr12	15035081	MGP	T	C	.	rs4236	0,35
chr16	16257016	ABCC6	G	A	.	rs63749794	0,00
chr16	16295863	ABCC6	T	C	.	rs72653762	0,0032
chr16	16248638	ABCC6	A	G	.	-	-
chr16	16251599	ABCC&	C	T	.	rs2238472	0,19
